# Prospective

**Published:** 1882-10

**Authors:** 


### Editorial.

### PROSPECTIVE.

In the initial number of the first volume of the REGISTER we recorded certain pledges concerning the conduct of the journal, and now, in this, the first number of volume two, we propose to reaffirm allegiance to the independent position which we then assumed, and to reiterate our intention of maintaining the same attitude.

We desire to have it distinctly understood that the REGISTER is, and shall continue to be, absolutely free from all merely local influences and from all entangling alliances of any kind whatsoever, and that its management is actuated solely by an earnest and honest desire to serve and benefit the medical profession, and is devoted to the promotion of the interests which all reputable medical men hold dear

In the accomplishment of these purposes we desire to have, and we hereby invoke, the aid and co-operation of our medical brethren everywhere. We now, cheerfully and most cordially, offer them the pages of the REGISTER as a medium of communication, one with another, and as a certain means, if properly employed, of insuring closer acquaintanceships and firmer and more enduring friendships among ourselves.

We shall always welcome, from any part of this State, or from other States, short or long accounts of cases of practical importance or of general interest; of local events pertaining to professional matters; as well as letters of inquiry, advice, instruction or information.

Every practitioner of medicine must necessarily encounter, in the regular rounds of his daily duties, experiences which should be reported and published; realizing the fact that thereby a fellow-worker, in perhaps a very remote field of labor, may be materially assisted, and the great cause of humanity advanced. More than this, by a general, we may say a promiscuous, aggregation of knowledge, the individual element, the personal bias, if you choose, which is inseparable from every single contribution, is gradually eliminated and truth is slowly but surely evolved.

This we regard an important mission, and one toward the accomplishment of which we shall most gladly contribute our humble share. We, therefore, ask our friends and our readers to consider this a direct personal appeal for such assistance in this direction as each may have in his power to render.

It is not the metropolitan practitioner alone who has the capacity, or who enjoys the opportunities for furnishing the contributions to which reference is herein made, but it is also to the country physician that we apply, and to whom the world must look for much of real, common-sense, practical advancement in medical science. History and experience have taught us this, and to day Jenner, Long, McDowell and a host of other glorious examples stand forth as proof of the assertion, and the very circumstances which surrounded these men, whose labors and whose unselfish contributions to the cause of science have made them immortal, obtain to-day. The opportunity is ours, let us not fail to improve it. Let us not refuse to subscribe our quota to the common fund of professional achievements.

In conclusion, we desire to state that it is our intention to add new features to the REGISTER, and to otherwise improve our journal as rapidly as its steadily increasing prosperity will warrant. It depends mainly upon our friends to determine how speedily these cherished objects shall be accomplished.

